# How the metabolic phenotype in adulthood is affected by long-lasting immunological trajectories since adolescence

**DOI:** 10.1038/s41598-022-13126-z

**Published:** 2022-05-31

**Authors:** Isaac Barroso, João Tiago Guimarães, Vanda Craveiro, Milton Severo, Elisabete Ramos

**Affiliations:** 1 Department of Clinical Pathology, São João Hospital Centre, EPE, Alameda Prof. Hernâni Monteiro, 4200-319 Porto, Portugal; 2 EPIUnit – Institute of Public Health, University of Porto, Porto, Portugal; 3 Department of Biomedicine, Faculty of Medicine, University of Porto, Porto, Portugal; 4 Department of Public Health and Forensic Sciences, and Medical Education, Faculty of Medicine, University of Porto, Porto, Portugal; 5 Laboratory for Integrative and Translational Research in Population Health (ITR), Porto, Portugal

**Keywords:** Inflammation, Biomarkers, Paediatric research

## Abstract

A close relationship between immune and metabolic systems has been perceived in the recent past. We aimed to assess whether the immunological trajectories of circulating white blood cells (WBC) started in adolescence, affects the metabolic phenotype in adulthood. We used data from 1183 participants of the population-based EPITeen cohort, evaluated at 13, 17, 21, 24 and 27 years of age. The Immunological trajectories from 13 to 27 years old were identified by mixed-effects models, being their association with metabolic features at 27 years old measured by logistic regression. The *Higher Inflammatory Activation trajectory* (*HIA trajectory*) had the highest percentage of individuals with metabolic syndrome, while *Lowest Levels of WBC trajectory* (*LLWBC trajectory*) showed the lowest percentage. Participants with *HIA trajectory* had significantly higher triglycerides, waist circumference, serum uric acid and BMI. After adjustment for sex and sports practice and *hs*-CRP, the odds of having one or more metabolic features in adulthood was significantly lower in *LLWBC trajectory*. Individuals with immunological trajectories of WBC linked with a pattern of higher immune activation showed a less favorable metabolic profile, while those with the lowest levels of WBC were less likely to have metabolic risk factors in adulthood.

## Introduction

Over the past twenty years, a close relationship between immune system and metabolism has become perceived, leading to emergence of the concept of immunometabolism, defined as the interplay between immunological and metabolic processes^1–3^. In fact, metabolic and immunological processes favor the development of a special environment in which the dysfunction of one system profoundly influences the other^4,5^.

The main findings supporting the link between immune function to metabolism arose when two independent studies found that the genetic absence of tumor necrosis factor (TNF) function led to a reduction in insulin resistance and impaired glucose tolerance in obese mouse lines^6,7^. In addition, as Hotamisligil reported, several immune mediators, when abnormally produced or regulated, contribute to the impairment of the metabolic function^8^. In this regard, inflammation has been implicated in the pathophysiology of the metabolic abnormalities^9,10^ and it has been demonstrated that metabolic stress may lead to impaired immune function, resulting in the so-called metaflammation^11,12^.

The peripheral circulating white blood cells (WBC) are established markers of immune response and have been associated with metabolic abnormalities, such as, obesity, insulin resistance, hypertension, dyslipidaemia and the progression or severity of atherosclerosis^4,13^. Therefore, total and differential WBC counts have been proposed as immune markers for predicting future cardiovascular events and mortality^14–16^. However, it remains unclear whether elevated WBC counts are directly involved in the deterioration of metabolic health or are just biomarkers of the ongoing inflammation^17^. Furthermore, it is not known whether the association of the WBC with the metabolic homeostasis is result of a chronic exposure or a consequence of acute metabolic alterations. In this sense, we aimed to assess whether immunological trajectories of WBC from adolescence to adulthood, were associated with features of metabolic syndrome in adulthood.

## Methods

### Subjects

The EPITeen cohort recruited in 2003–2004 the adolescents born in 1990 that were enrolled in public and private schools in Porto, Portugal. At baseline, 2786 eligible participants were identified and 2159 agreed to participate, resulting in an overall participation rate of 77.5%. In the second wave, 1716 (79.5%) of the baseline participants were re-evaluated at 17 years and 783 new participants were integrated in the cohort since they moved to a school in Porto. From the entire cohort, 1764, 1094 and 1244 participants were re-evaluated at 21, 24 and at 27 years, respectively. In all waves, WBCs measurements from evaluations with concentrations of high-sensitivity C-reactive protein (*hs*-CRP) ≥ 10 mg/L were excluded (n = 15) since it constitutes a marker of acute infection ^18^. We included participants having valid WBCs measurements in at least 3 of the 5 evaluations. The final sample to define the immunological trajectories consisted of 1183 subjects. To study its association with metabolic syndrome at age 27, 276 individuals were excluded due to missing values for any of the features. Thus, the final sample was composed by 907 participants.

Included participants had lower proportion of individuals with obesity, higher proportion of chronic medication intake, higher parental education and higher proportion of sports practice, however, they were less active during leisure-time (Table A1).

### Data collection

In the first two study waves, participants were assessed in schools and, in the subsequent assessments, they were invited to come to our University Department. Data collection was performed through self-administered and face-to-face questionnaires answered by the participants at school (behavioral characteristics) and their parents at home (social and demographic characteristics, as well as individual and familiar disease history). Sports practice was a yes-or-no question answered by adolescents, considering the practice of sports in addition to the compulsory school curriculum, regardless of frequency or intensity. Data from evaluation at 17 years was used when there were missing values for practice of sports at 13 years.

A physical examination consisted of anthropometric measurements and a fasting blood sample collection. All procedures were performed by a team of trained healthcare professionals and standardized over time.

### Physical examination

Weight and height were obtained with the subject in light indoor clothes and barefoot. Weight was measured in kilograms, to the nearest tenth, using a digital scale (Tanita TBF-300, Tanita Corporation of America,Inc., Illinois, USA) and height was measured in centimeters, to the nearest tenth, using a portable stadiometer (stadiometer 213I, Seca GmbH, Hamburg, Germany). Based on these two parameters, the body mass index (BMI) was calculated.

Waist circumference was measured to the nearest centimeter, mid-way between the lower limit of the rib cage and the iliac crest, with the subject standing, using a flexible and non-distensible tape and avoiding exertion of pressure on the tissues.

Blood pressure (BP) was measured using an oscillometric method (OMRON® Blood Pressure Monitor, M6 Comfort), according to standardized procedures^19^. After at least 10 min of rest, two BP measurements were taken, separately by at least five minutes; a third measure was taken if the difference between the first two was higher than 5 mmHg. The average of the two closest measurements was used in the analysis.

### Blood measurements

A venous blood sample was drawn after an 8-h overnight fast. All the samples were analyzed at the central laboratory of *São João University Hospital Centre*. Total and differential WBC counts (neutrophils, monocytes, lymphocytes, eosinophils and basophils) were obtained using an automated blood counter Sysmex®XE-5000 (Hyogo, Japan). The *hs*-CRP was determined through particle-enhanced immunonephelometry using an auto-analyzer Behring, Nephelometer II® (Dade Behring Marburg GMBH, Germany).

Serum glucose and uric acid, total cholesterol, triglycerides, high-density lipoprotein (HDL)-cholesterol and low-density lipoprotein (LDL)-cholesterol were assessed using a Beckman Coulter AU5400 analyzer. Serum insulin was measured using a ^125^I-labelled insulin radioimmunoassay method. Insulin resistance was estimated according to the homeostatic model assessment (HOMA), as the product of fasting glucose (mmol/L) and insulin (μUI/mL) divided by a constant 22.5^20^.

### Covariates

Practice of sports was a yes-or-no question considering the practice of sports in addition to the compulsory school curriculum, regardless of frequency or intensity. Data from evaluation at 17 years was used when there were missing values for practice of sports at 13 years.

### Metabolic syndrome

Metabolic syndrome was defined according to the American Heart Association (AHA)/National Heart, Lung, and Blood Institute (NHLBI) guidelines^21^. Thus, Metabolic syndrome is defined as the presence of at least 3 of the following 5 criteria: waist circumference ≥ 94 cm in males and ≥ 80 cm in females; HDL cholesterol ˂ 40 mg/dL in males and ˂ 50 mg/dL in females; triglycerides ≥ 150 mg/dL; systolic blood pressure (SBP) ≥ 130 and/or diastolic (DBP) ≥ 85 mmHg; fasting glucose ≥ 100 mg/dL.

### Leukocyte trajectories

To have a summary measure on the exposure overtime, as well as to reduce the random error associated at each measurement, we classified participants according to the pattern of evolution of the WBCs from 13 to 27 years old. Individual trajectories for total and differential WBCs were evaluated and participants were allocated into six clusters^22^. One of the clusters was composed only by 17 participants and was not considered for further analyses since they were considered probable outliers due to extreme values in at least one of the evaluations. Thus, our final analysis included 1166 participants with a leukocyte distribution trajectory identified. The leukocyte trajectories were named according to a distinctive characteristic, except for one that was named as undefined: *Higher Inflammatory Activation (HIA)*; *Lowest Levels of WBC (LLWBC)*; *Highest Proportion of Eosinophils (HPEo)*; *Lowest Proportion of Eosinophils (LPEo)*; *Undefined (Un)*. Overall, subjects in *HIA trajectory* (n = 136) had the highest total WBC count and percentage of neutrophils, as well as the lowest percentage of lymphocytes, while participants in *LLWBC trajectory* (n = 285) had the lowest total WBC count without significant differences regarding the distribution of the differential WBCs. The participants in the *HPEo trajectory* (n = 238) showed the highest eosinophils proportion and lowest percentage of neutrophils, while those in *LPEo trajectory* (n = 344) had the lowest percentage of eosinophils, basophils and monocytes, as well as the highest lymphocyte proportion. Participants in *Un trajectory* (n = 163) showed the highest percentage of monocytes and basophils.

### Ethical considerations

The EPITeen cohort was conducted according to the 1964 Declaration of Helsinki guidelines and its later amendments. The Ethics Committee of the *São João University Hospital Centre* and the Ethics Committee of the Institute of Public Health from the University of Porto approved this project, and appropriate standard procedures were developed to guarantee data confidentiality and protection. Written and oral information explaining the purpose and design of the study was given to the adolescents and parents/legal guardians, and signed written informed consent was obtained from both at 13 and 17 years, and only from participants at 21, 24 and 27 years. Thus, written informed consent was obtained from all subjects and/or their legal guardian(s).

### Statistical analysis

To identify the individual trajectories for total and differential WBCs we started by testing if the variables (total WBC and each of the sub-types) had a linear, quadratic or cubic trend with time; we found that, except basophiles, which were linear, the other ones were quadratic. Then, to extract the individual trajectories in time for each variable several parameterizations of mixed effects models^23^ were tested by including step by step random intercept, slope, and quadratic terms. We used finite Gaussian mixture models^24^ to identify the clusters of individual trajectories with the same characteristics within clusters, and different characteristics between clusters. We used the random effects extracted in the previous step from the final mixed model as variables in mixture models. The process was performed into two steps. The first step was the capture of individual’s differences compare to the population trend by extracting the random effects. The second step consisted in the applying of mixture Gaussian models to the random effects extracted, so that the cluster was built independently of the first step. Finally, the number of clusters (leukocyte trajectories) was chosen according to the Bayesian Information Criterion (BIC), assuming the lowest the BIC as the better solution (Fig. A1). The median individual posteriori probabilities for the most likely trajectory (the one that was used to classify the participants) ranged from 78 to 99%, showing a very low probability to have misclassification. We used intraclass correlation coefficient (ICC) to evaluate how much a value at each moment agrees with the remaining for the same participant. The ICC regarding the mean values of each WBC subtypes was: total WBC—0.80; neutrophils—0.75; eosinophils—0.86; basophils—0.77; lymphocytes—0.76; monocytes—0.85. These high correlations show a great stability of the trajectory over time.

Chi-square test was used to compare proportions. Means and standard-deviations (SDs) were compared using Student’s *t*-test or ANOVA, as appropriate. Since HOMA-IR and *hs*-CRP parameters followed a non-parametric distribution, they were presented by median (25th–75th percentiles) and compared through Kruskal–Wallis test. Associations between the leukocyte trajectories and the presence of at least one metabolic feature were estimated using odds ratio (OR) and 95% confidence intervals (95% CI) by logistic regression. The results were adjusted for sex (model 1), and for sex and sports practice (model 2). The selection of confounders was based on the characteristics that are different between trajectories^22^ and rely on prior knowledge that support whether those variables could also be a cause for metabolic features. Additionally, we performed a model adjusting to *hs*-CRP levels (model 3), in order to evaluate a possible mediator effect.

Statistical significance was considered with a significant level of 0.05. The packages nlme and mclust from the R software v.3.6.0 were used to estimate the mixed effects models and the finite Gaussian mixture models, respectively^25^. Other statistical analysis was performed using IBM® SPSS® Statistics version 25.0 (IBM Corp., Armonk, NY, USA).

### Ethical consent statement

 Written informed consent was obtained from all subjects and/or their legal guardian(s).

## Results

The prevalence of each metabolic syndrome feature is shown in the Table 1. Globally, the most common feature was high waist circumference (21.8%), the lowest frequent was high fasting glucose (2.6%). Waist circumference is the only feature which prevalence had statistically significant differences between trajectories with *HIA trajectory* showing the highest prevalence (28.9%) and *LLWBC trajectory* the lowest (15.1%). The prevalence of metabolic syndrome (≥ 3 features) was 3.0% and was higher among those in the *HIA trajectory* (5.2%), while those in the *LLWBC* and *Un trajectories* showed the lowest prevalence (2.3%)*.* None of the participants had the five features.

**Table 1:** Prevalence of each metabolic syndrome feature and number of features according to Leukocyte trajectories.

Metabolic features	Trajectory/N (%)						*P* value^a^
	Total	*LPEo* trajectory	*HPEo* trajectory	*LLWBC* trajectory	*HIA* trajectory	*Un* trajectory	
		n = 267	n = 191	n = 221	n = 97	n = 131	
**Waist circumference**							**0.029**
< 94 cm in males and < 80 cm in women	705 (78.2)	205 (77.4)	141 (74.2)	186 (84.9)	69 (71.1)	104 (80.0)	
≥ 94 cm in males and ≥ 80 cm in women	196 (21.8)	60 (22.6)	49 (25.8)	33 (15.1)	28 (28.9)	26 (20.0)	
**Fasting glucose**							0.881
< 100 mg/mL	882 (97.4)	262 (98.1)	185 (96.9)	213 (96.8)	94 (96.9)	128 (97.7)	
≥ 100 mg/mL	24 (2.6)	5 (1.9)	6 (3.1)	7 (3.2)	3 (3.1)	3 (2.3)	
**Triglycerides**							0.099
< 150 mg/mL	838 (92.6)	245 (91.8)	175 (91.6)	210 (95.9)	85 (87.6)	123 (93.9)	
≥ 150 mg/mL	67 (7.4)	22 (8.2)	16 (8.4)	9 (4.1)	12 (12.4)	8 (6.1)	
**HDL-C**							0.934
≥ 40 mg/dL in males and ˃ 50 mg/dL in women	787 (87.0)	234 (87.6)	164 (85.9)	193 (88.1)	84 (86.6)	112 (85.5)	
< 40 mg/dL in males and < 50 mg/dL in women	118 (13.0)	33 (12.4)	27 (14.1)	26 (11.9)	13 (13.4)	19 (14.5)	
**Blood pressure**							0.764
Systolic < 130 and/or diastolic < 85 mmHg	795 (87.7)	233 (87.6)	165 (86.4)	199 (90.0)	83 (85.6)	115 (87.8)	
Systolic ≥ 130 and/or diastolic ≥ 85 mmHg	111 (12.3)	33 (12.4)	26 (13.6)	22 (10.0)	14 (14.4)	16 (12.2)	
**No of metabolic features**							0.126
0	543 (59.0)	158 (59.2)	106 (55.5)	153 (69.2)	51 (52.6)	75 (57.3)	
1	244 (26.9)	73 (27.3)	54 (28.3)	44 (19.2)	29 (29.9)	44 (33.6)	
2	93 (10.3)	29 (10.9)	24 (12.6)	19 (8.6)	12 (12.4)	9 (6.9)	
≥ 3	27 (3.0)	7 (2.6)	7 (3.7)	5 (2.3)	5 (5.2)	3 (2.3)	

LPEo: Lowest proportion of eosinophils; HPEo: highest proportion of eosinophils; LLWBC: Lowest levels of WBC; HIA: higher inflammatory activation; Un: Undefined. *P*-values < 0.05 are in bold.

^a^Chi-square test was used to compare proportions.

The Table 2 describes the metabolic related parameters according to the trajectories. In general, participants belonging to the *HIA trajectory* presented the worse cardiometabolic profile with significantly higher levels of triglycerides [mean (standard deviation)] [96.4 (46.9) mg dL^−1^], waist circumference [84.2 (12.3) cm], BMI [24.8 (4.9) Kg m^2^] and serum uric acid [5.03 (1.35) mg dL^−1^]. On the other hand, those belonging to the *LPEo trajectory* showed significantly higher values of total and HDL cholesterol [180.5 (35.4) mg dL^−1^, and 58.0 (12.9) mg dL^−1^, respectively]. Participants belonging to the *LLWBC trajectory* had significantly lower values of *hs*-CRP [median (25th–75th percentiles)] [0.85 (0.50–2.10)].

**Table 2:** Metabolic related parameters according to the Leukocyte trajectories.

Metabolic parameters	*LPEo* trajectory	*HPEo* trajectory	*LLWBC* trajectory	*HIA* trajectory	*Un* trajectory	*P*-value^a^
	Mean (SD)	Mean (SD)	Mean (SD)	Mean (SD)	Mean (SD)	
Fasting glucose (mg/dL)	84.0 (8.2)	86.2 (28.8)	85.6 (7.5)	86.1 (7.5)	83.8 (7.2)	0.389
HDL-C (mg/dL)	58.0 (12.9)	54.2 (11.9)	54.2 (12.8)	55.0 (13.6)	55.3 (12.1)	**0.006**
Triglycerides (mg/dL)	89.3 (40.0)	83.3 (37.4)	79.0 (45.6)	96.4 (46.9)	88.0 (61.1)	**0.013**
SBP (mmHg)	111.8 (12.6)	113.6 (11.9)	114.0 (11.9)	115.4 (12.2)	113.6 (12.3)	0.100
DBP (mmHg)	70.8 (8.1)	71.1 (7.7)	70.4 (7.3)	71.7 (8.1)	70.4 (7.9)	0.672
Waist circumference (cm)	79.8 (11.6)	81.5 (11.3)	81.3 (9.9)	84.2 (12.3)	79.4 (9.1)	**0.004**
Total colesterol (mg/dL)	180.5 (35.4)	172.0 (32.2)	172.3 (30.1)	179.3 (36.8)	174.5 (32.4)	**0.023**
LDL-C (mg/dL)	104.9 (28.6)	101.0 (26.3)	102.6 (24.7)	105.0 (30.0)	101.6 (26.7)	0.524
Uric Acid (mg/dL)	4.65 (1.27)	4.92 (1.21)	4.99 (1.20)	5.03 (1.35)	4.72 (1.21)	**0.008**
BMI (kg/m^2^)	23.5 (4.2)	23.8 (4.2)	23.6 (3.5)	24.8 (4.9)	23.2 (3.6)	**0.039**

	Median (P25–P75)	Median (P25–P75)	Median (P25–P75)	Median (P25–P75)	Median (P25–P75)	
HOMA-IR	1.69 (1.28–2.28)	1.44 (1.07–1.91)	1.54 (1.10–2.04)	1.62 (1.27–2.39)	1.44 (1.15–2.08)	**< 0.001**
*hs*-CRP (mg/L)	1.40 (0.60–3.70)	0.95 (0.40–2.20)	0.85 (0.50–2.10)	1.20 (0.50–3.30)	1.00 (0.50–3.70)	**< 0.001**

LPEo: Lowest proportion of eosinophils; HPEo: highest proportion of eosinophils; LLWBC: lowest levels of WBC; HIA: higher inflammatory activation; Un: undefined. HOMA-IR: homeostatic model assessment of insulin resistance; HDL-C: high-density lipoprotein cholesterol; LDL-C: low-density lipoprotein colesterol; SBP: systolic blood pressure; DBP: diastolic blood pressure; BMI: body mass index; *hs*-CRP: *high-sensitivity* C-reactive protein. P25–P75: 25th–75th percentiles. *P*-values < 0.05 are in bold.

^a^ANOVA test was used to compare means and standard-deviations (SD), while Kruskal–Wallis test was used to compare medians (P25–P75).

The association between leukocyte trajectories and metabolic features is depicted in Table 3. Using *LPEo trajectory* as reference, participants in the *HIA trajectory* presented the higher odds to have at least a metabolic syndrome feature, although without reach statistical significance [OR = 1.31, 95% CI: 0.82, 2.09]. On the contrary, individuals in the *LLWBC trajectory* showed significantly lower odds of having one or more metabolic features in adulthood [OR = 0.64, 95% CI: 0.44, 0.94]. Similar results were found after adjusting for sex and sports practice. To assess whether inflammation is a mediator in the association between immunological trajectories and metabolic outcomes, we additionally adjusted for *hs*-CRP and no changes were observed.

**Table 3:** Association between Leukocyte trajectories and at least one metabolic feature (≥ 1 vs. 0).

	Crude	Adjusted for sex	Adjusted for sex and sports practice	Adjusted for sex and sports practice and *hs*-CRP
		Model1	Model2	Model3
	≥ 1 Metabolic feature	≥ 1 Metabolic feature	≥ 1 Metabolic feature	≥ 1 Metabolic feature
*LPEo* trajectory	Reference	Reference	Reference	Reference
*HPEo* trajectory	1.16 (0.80, 1.69)	1.14 (0.78, 1.68)	1.17 (0.80, 1.72)	1.26 (0.85, 1.88)
*LLWBC* trajectory	**0.64 (0.44, 0.94)**	**0.63 (0.43, 0.93)**	**0.64 (0.44, 0.95)**	**0.68 (0.46, 1.00)**
*HIA* trajectory	1.31 (0.82, 2.09)	1.29 (0.81, 2.07)	1.23 (0.76, 1.97)	1.21 (0.74, 1.97)
*Un* trajectory	1.08 (0.71, 1.65)	1.08 (0.70, 1.64)	1.07 (0.70, 1.64)	1.07 (0.69, 1.65)

LPEo: Lowest proportion of eosinophils; HPEo: highest proportion of eosinophils; LLWBC: lowest levels of WBC; HIA: higher inflammatory activation; Un: undefined. CI: confidence interval; OR: odds ratio. Statistically significant estimates are in bold.

## Discussion

In this study, we found that individuals with immunological trajectories of WBC associated with a pattern of higher immune activation had higher odds to have a worse metabolic profile while those with immunological trajectories associated with an immunoprotective response pattern showed lower odds of having a worse metabolic profile. To the best of our knowledge this is the first study to assess the relationship between the longitudinal exposure to an immunological pattern and the metabolic phenotype. These results support that the response of the immune system have an impact on the metabolic profile.

In this study, individuals belonging to *HIA trajectory* with immunological trajectories of WBC associated with a pattern of immune activation (highest total WBC count and percentage of neutrophils, as well as the lowest percentage of lymphocytes) showed a less favorable metabolic profile, with the highest percentage of metabolic syndrome (though a very small percentage). Our results are in accordance with previous reports that highlight a close relationship between immune and metabolic functions, sometimes referred as the immunometabolic crosstalk, in which immune responses directly influence metabolic function, with inflammation associated with deleterious effects on the metabolic homeostasis^15,26,27^. In this sense, the observed association between *HIA trajectory* with a less favorable metabolic profile could be explained, at least in part, by increased inflammation status of these individuals since several immune mediators, such as TNF-α, MCP-1 and IL-6, may play a pivotal role to induce resistance insulin through the overactivation of various inflammatory pathways in liver, muscle and adipose tissue, whereas its downregulation results in reduced adiposity and improved insulin sensitivity^2,28^. Moreover, some studies point out that a high total WBC count may cause a chronic low-grade inflammation that will impair endothelial function^29^. In line with this hypothesis, individuals belonging to the *HIA trajectory* showed higher serum uric acid levels^13,21,30^. Increased serum uric acid concentrations have been independently associated with impaired metabolic function^13,31^ and, since this parameter was not considered in the definition of the trajectories, these results support the relationship between the activation of the immune system and the compromised metabolic profile. However, despite inflammation could explain part of the association between the *HIA trajectory* and a less favorable metabolic profile, since these results persisted even after adjusting for *hs*-CRP, other mechanisms could intervene in the regulation of this association and should be addressed in future research studies.

On the other hand, those individuals belonging to the *LLWBC trajectory* (with lowest total WBC count) are associated with an immunoprotective response pattern^32,33^, will have a lower low-grade inflammation that may, at least partially, explain the better metabolic function and the significantly lower odds of having metabolic features associated with metabolic syndrome. In fact, these individuals had the highest percentage of sports practice, and previous studies have shown that high levels of physical activity are associated with reduced systemic inflammation and total WBC levels in both men and women, regardless of initial weight^33,34^. Interestingly, this association persisted even after adjusting for sports practice, which may be explained by the effect of other related social and environmental exposures that are intrinsically linked to the immune system response^35–38^.

In addition, despite women having a greater immune responsiveness due to sex hormones, especially estrogens, and mounting stronger pro-inflammatory responses^39,40^, in this study the results were similar by sex.

The assessment of the relationship between immunological trajectories of WBC and the metabolic syndrome may help us to understand the pathophysiology of this condition, since subclinical, low-grade inflammation has been recognized as a key player in its onset^41,42^. Since no changes were observed after adjustment for *hs*-CRP, inflammation may not be an intermediate step in the association between immunological trajectories and the metabolic outcomes, and other underlying mechanisms may play a role in this association. However, we cannot exclude that long-term exposure to low-grade inflammation may have contributed to the creation of immunological trajectories or that these trajectories and the levels of inflammation may have a common cause that precedes them. The role of inflammation as a mediator in the association between immunological trajectories and the metabolic outcomes should be studied when there is a higher incidence of metabolic alterations, which will allow the use of more appropriate methodologies to better understand it.

Since we reported long-lasting immunological trajectories of WBC that are stable from adolescence to adulthood, a strength of this work is that the trajectories were developed in a relatively healthy life-period, which suggests that the associations found here are not conditioned by metabolic abnormalities resulting from underlying chronic pathologies or medications.

Nevertheless, this study should be interpreted in light of some limitations. The absence of leukocyte immunophenotyping does not allow the evaluation of specific WBC subsets and, therefore, hinders a deeper characterization of the studied population. Furthermore, to obtain a deeper characterization of the relationship between the immune system and metabolic function, other immune mediators assessing pro-inflammatory pathways could also have been assessed. Some participants were lost over time, as well as at the beginning due to refusal to give a blood sample. Included participants tend to have a healthier profile than excluded participants (higher proportion of individuals practicing sports and lower percentage of obese subjects). Although this selection bias may prevent knowing the prevalence of each trajectory, it does not change its characteristics. Additionally, the bias may have an impact on hiding other potential trajectories, since it contributes to creating a more homogenous sample than the population. Furthermore, the age profile of our sample also contributes to weakening the association, since at the age of 27, a pronounced metabolic dysfunction is not expected to be observed.

On the other hand, the longitudinal approach of a healthy sample is one of the strengths of this study, since most results are from cross-sectional studies and with older participants^43,44^. In addition, the 14-year of follow-up makes this study a unique opportunity to longitudinally understand a crucial period of life – the transition from adolescence to adulthood. Lastly, since at the age of 27 it is usually too early for the onset of chronic diseases, most participants do not undergo pharmacological therapy, which could have compromised the findings of the study. Therefore, our results are one of most prone to support causality and may be fairly helpful for stating future research hypotheses. Actually, the observational nature of the study does not allow to ensure causality, since there are uncontrolled variables that may interfere with the results. However, the longitudinal nature of the study ensures the temporal relationship between exposure and outcome, which is a strong indicator of a causal relationship.

In conclusion, for the first time we reported that trajectories of WBC, stable from adolescence to adulthood, are associated with the metabolic profile in adulthood. We observed that individuals with trajectories linked with a pattern of higher immunological activation had a less favorable metabolic profile, while those with immunological trajectories of WBC linked with an immunoprotective pattern of response were less likely to have metabolic risk factors in adulthood. Since our study focus on immunological trajectories started during adolescence, these results highlight the immunological potential to a long-term effect on the metabolic homeostasis. Further research is mandatory to better characterize the influence of the immune system on metabolic homeostasis and, ultimately, to promote early interventions that favor metabolic health in adulthood.

## Supplementary Information

Supplementary Information.

## Data Availability

The datasets used and/or analysed during the current study available from the corresponding author on reasonable request.
