# Supplementary material for: How the metabolic phenotype in adulthood is affected by long-lasting immunological trajectories since adolescence
Source: Sci Rep. 2022 May 31;12:9085. doi: 10.1038/s41598-022-13126-z (PMC9156771; doi:10.1038/s41598-022-13126-z)

**Additional Files**

**Table A1** – Description and comparison of the characteristics at baseline of non-included and included participants in the present analysis.

|  | **Non-Included** | | **Included** | | **p-value** |
| --- | --- | --- | --- | --- | --- |
| **Characteristics** | **n** | **%** | **n** | **%** |  |
|  | 1776 | 60.4 | 1166 | 39.6 |  |
| **Sex** |  |  |  |  | 0.407 |
| Female | 921 | 51.9 | 586 | 50.3 |  |
| **Chronic disease** |  |  |  |  | 0.308 |
| Yes | 206 | 19.0 | 187 | 20.9 |  |
| Missing | 690 |  | 271 |  |  |
| **Allergic disease** |  |  |  |  | 0.591 |
| Yes | 341 | 33.1 | 295 | 34.3 |  |
| Missing/Did not know | 745 |  | 306 |  |  |
| **Allergy** |  |  |  |  | 0.496 |
| Yes | 265 | 25.7 | 234 | 27.2 |  |
| Missing/Did not know | 746 |  | 306 |  |  |
| **Asthma** |  |  |  |  | 0.720 |
| Yes | 118 | 11.5 | 104 | 12.1 |  |
| Missing/Did not know | 749 |  | 303 |  |  |
| **Rhinitis** |  |  |  |  | 0.313 |
| Yes | 94 | 9.3 | 92 | 10.8 |  |
| Missing/Did not know | 768 |  | 315 |  |  |
| **Chronic medication** |  |  |  |  | **0.001** |
| Yes | 522 | 51.8 | 516 | 59.4 |  |
| Missing | 768 |  | 297 |  |  |
| **Parental education** |  |  |  |  | **<0.001** |
| ≤6 years | 553 | 31.3 | 221 | 19.0 |  |
| 7-9 years | 364 | 20.5 | 199 | 17.1 |  |
| 10-12 years | 399 | 22.5 | 332 | 28.5 |  |
| >12 years | 350 | 21.0 | 411 | 35.3 |  |
| Missing | 110 |  | 3 |  |  |
| **Practice of sports** |  |  |  |  | **<0.001** |
| Yes | 440 | 44.9 | 481 | 55.7 |  |
| Missing | 797 |  | 302 |  |  |
| **Leisure-time physical activity** |  |  |  |  | **<0.001** |
| Mainly sitting | 242 | 25.4 | 267 | 32.5 |  |
| Mainly standing | 227 | 23.8 | 168 | 20.5 |  |
| Active | 255 | 26.8 | 275 | 33.5 |  |
| Very active | 229 | 24.0 | 111 | 13.5 |  |
| Missing | 823 |  | 345 |  |  |
| **Body mass index** |  |  |  |  | **0.025** |
| Underweight | 19 | 1.7 | 9 | 1.0 |  |
| Normal weight | 783 | 69.3 | 641 | 70.7 |  |
| Pre-obesity | 212 | 18.8 | 193 | 21.3 |  |
| Obesity | 116 | 10.3 | 64 | 7.1 |  |
| Missing | 646 |  | 259 |  |  |

**Legend:** P-values <0.05 are in bold.

**Figure A1** – Changes on Bayesian Information Criterion (BIC) according to the number of clusters.


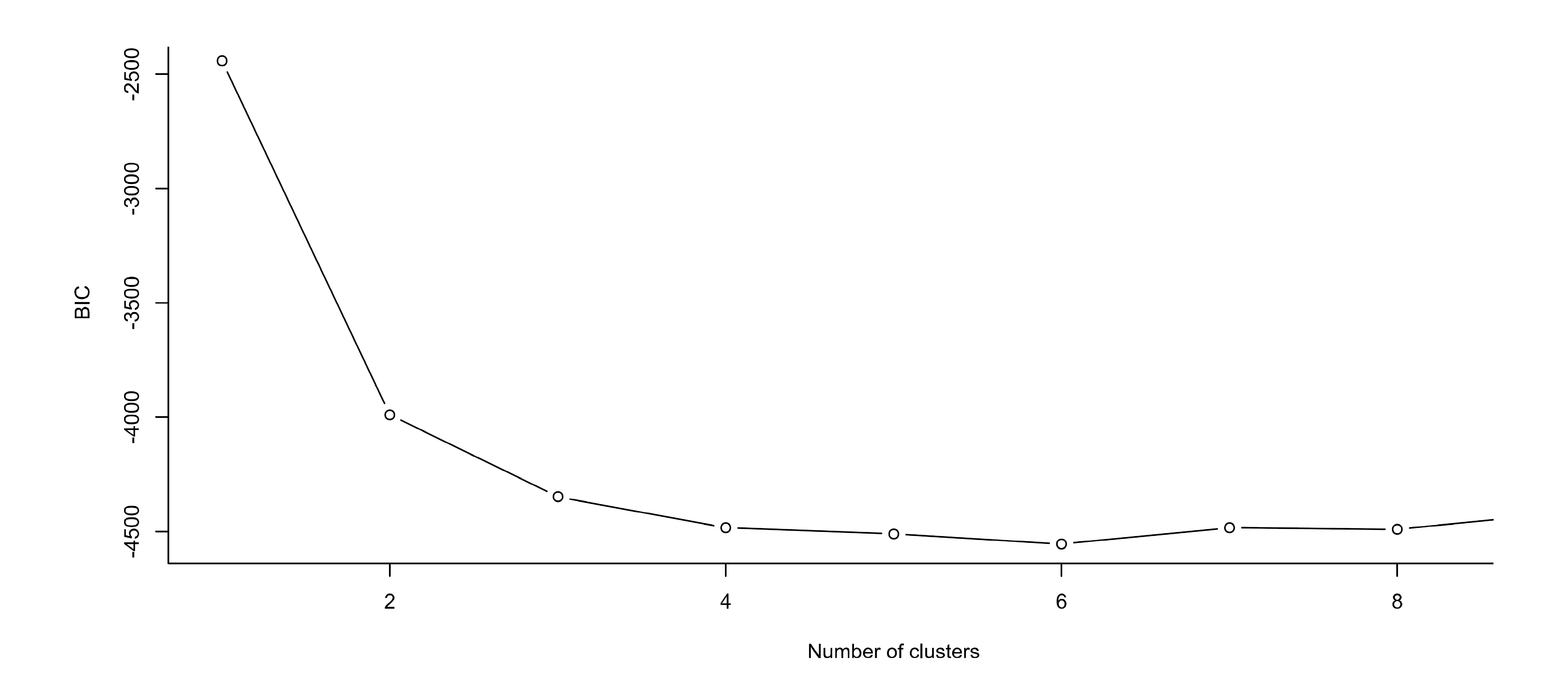

Supplement: Supplementary file 1 — Supplementary Information. [file 41598_2022_13126_MOESM1_ESM.docx]
